# Histopathological and immunological characteristics of placentas infected with chikungunya virus

**DOI:** 10.3389/fmicb.2022.1055536

**Published:** 2022-11-17

**Authors:** Natália Salomão, Kíssila Rabelo, Elyzabeth Avvad-Portari, Carlos Basílio-de-Oliveira, Rodrigo Basílio-de-Oliveira, Fátima Ferreira, Luiz Ferreira, Thiara Manuele de Souza, Priscila Nunes, Monique Lima, Anna Paula Sales, Regina Fernandes, Luiz José de Souza, Laura Dias, Patrícia Brasil, Flavia dos Santos, Marciano Paes

**Affiliations:** ^1^Laboratório Interdisciplinar de Pesquisas Médicas, Instituto Oswaldo Cruz, Fundação Oswaldo Cruz, Rio de Janeiro, Brazil; ^2^Laboratório de Imunologia Viral, Instituto Oswaldo Cruz, Fundação Oswaldo Cruz, Rio de Janeiro, Brazil; ^3^Laboratório de Ultraestrutura e Biologia Tecidual, Universidade do Estado do Rio de Janeiro, Rio de Janeiro, Brazil; ^4^Departamento de Anatomia Patológica, Instituto da Mulher e da Criança Fernandes Figueira, Fundação Oswaldo Cruz, Rio de Janeiro, Brazil; ^5^Departamento de Anatomia Patológica, Universidade Federal do Estado do Rio de Janeiro, Rio de Janeiro, Brazil; ^6^Departamento de Neonatologia, Universidade Federal do Estado do Rio de Janeiro, Rio de Janeiro, Brazil; ^7^Departamento de Anatomia Patológica, Instituto Nacional de Infectologia Evandro Chagas, Fundação Oswaldo Cruz, Rio de Janeiro, Brazil; ^8^Laboratório Estratégico de Diagnóstico Molecular, Instituto Butantan, São Paulo, Brazil; ^9^Centro de Referência de Doenças Imuno-infecciosas (CRDI), Campos dos Goytacazes, Rio de Janeiro, Brazil; ^10^Faculdade de Medicina de Campos, Campos dos Goytacazes, Rio de Janeiro, Brazil; ^11^Laboratório de Biotecnologia, Universidade Estadual do Norte Fluminense, Campos dos Goytacazes, Rio de Janeiro, Brazil; ^12^Hospital Geral Dr. Beda, CEPLIN – Uti Neonatal Nicola Albano, Campos dos Goytacazes, Rio de Janeiro, Brazil; ^13^Laboratório de Doenças Febris Agudas, Instituto Nacional de Infectologia Evandro Chagas, Fiocruz, Rio de Janeiro, Brazil

**Keywords:** placenta, chikungunya, histopathology, immunohistochemistry, cytokines

## Abstract

Although vertical transmission of CHIKV has been reported, little is known about the role of placenta in the transmission of this virus and the effects of infection on the maternal-fetal interface. In this work we investigated five placentas from pregnant women who became infected during the gestational period. Four formalin-fixed paraffin-embedded samples of placenta (cases 1–4) were positive for CHIKV by RT-PCR. One (case 5) had no positive test of placenta, but had positive RT-PCR for CHIKV in the serum of the mother and the baby, confirming vertical transmission. The placentas were analyzed regarding histopathological and immunological aspects. The main histopathological changes were: deciduitis, villous edema, deposits, villous necrosis, dystrophic calcification, thrombosis and stem vessel obliteration. In infected placentas we noted increase of cells (CD8^+^ and CD163^+^) and pro- (IFN-γ and TNF-α) and anti-inflammatory (TGF-β and IL-10) cytokines compared to control placentas. Moreover, CHIKV antigen was detected in decidual cell, trophoblastic cells, stroma villi, Hofbauer cells, and endothelial cells. In conclusion, CHIKV infection seems to disrupt placental homeostasis leading to histopathological alterations in addition to increase in cellularity and cytokines overproduction, evidencing an altered and harmful environment to the pregnant woman and fetus.

## Introduction

Chikungunya virus (CHIKV) is an arbovirus of the *Togaviridae* family and *Alphavirus* genus (Schuffenecker et al., 2006), which was first isolated in 1953 in Tanzania. It is endemic in Africa and Asia (Lumsden, 1955); however in 2014, it was introduced in the Americas, causing major epidemics (Escobar et al., 2016). CHIKV is a positive-strand RNA virus, enveloped, and spherical with a 60–70 nm-diameter (Higashi et al., 1967; Simizu et al., 1984; Powers et al., 2001). Its name is derived from a Makonde word meaning “the one which bends up” due to the posture some infected individuals assume as a result of arthralgia (Mavalankar et al., 2008). Other common symptoms are fever, rash, headache and myalgia (Silva and Dermody, 2017).

The principal mode of CHIKV transmission is by the bite of infected *Aedes* mosquitoes. However, although rare, CHIKV vertical transmission has already been reported, with the first evidence in June 2005, during an outbreak on La Reunion Island (Robillard et al., 2006). As consequence, symptomatic neonates may develop classic chikungunya similar to adults, which resolves spontaneously within 2 weeks (Ramful et al., 2007). Nevertheless, complicated or severe forms of chikungunya, such as hemorrhagic, cardiac, and neurologic manifestations have also been reported in neonates (Gérardin et al., 2008; Senanayake et al., 2009), requiring admission to intensive care units, intubation, and mechanical ventilation (Ramful et al., 2007). The rate of infected newborn was up to 49%, in mothers with intrapartum viremia (within 2 days of delivery; Ramful et al., 2007), thus, the intrapartum period seems to be the most critical one for transmission to the neonate. Most authors suggest transplacental transmission shortly before delivery is the most likely mode of transmission *via* mother-to-child through microtransfusions when the mother is experiencing CHIKV viremia (Ramful et al., 2007). Early maternal-fetal transmission of CHIKV has also been reported. Spontaneous abortion was observed in three cases of CHIKV infection before 16 weeks of gestation, and the viral genome was detected in amniotic fluid, chorionic villi, and in the fetal brain (Lenglet et al., 2006; Touret et al., 2006). There have also been reports of spontaneous abortion associated with CHIKV infection in which the virus was detected and placental samples exhibited malformations (Salomão et al., 2021). The presence of CHIKV has been reported in the placenta (Prata-Barbosa et al., 2018), newborn cerebrospinal fluid, amniotic fluids (Grivard et al., 2007), serum (Shenoy and Pradeep, 2012; Lyra et al., 2016), and urine (Lyra et al., 2016) along with Chikungunya IgM antibody in cerebrospinal fluid.

Here, we aimed to analyze placentas of five women who had confirmed Chikungunya during pregnancy, focusing on histopathology, investigation of viral antigens, as well as cells and cytokines that may have contributed to placental alterations.

## Materials and methods

### Ethical procedures

The study was approved by the Ethics Committee of the Oswaldo Cruz Foundation (CAEE: 92728218.5.0000.5248). All the mothers provided written informed consent and authorized publication of the results.

### Case descriptions

The pregnant women (PW) became infected between 2018 and 2019 during Chikungunya epidemics in the cities of Rio de Janeiro and Campos dos Goytacazes. In 2019, incidence was 374.05 cases per 100,000 inhabitants in Rio de Janeiro (Superintendência de Vigilância em Saúde, 2018; CGVS, 2019). In 2018, incidence was 918.9 cases per 100,000 in Campos dos Goytacazes and rose to 942.5 cases per 100,000 in the first 6 months of 2019 (Secretaria de estado de saúde do Rio de Janeiro, 2019). The cases are described below:

– Case 1: A 40-year-old patient at 36 weeks of gestation (3^rd^ trimester). She attended the Regional Center for Infectious Diseases (CRDI) of the Plantadores de Cana Hospital (Campos dos Goytacazes) in May 2018, reporting symptoms such as headache, severe arthralgia and exanthema. Three days after the onset of symptoms, she was hospitalized with oligohydramnios. Serological tests were performed during 3^rd^ trimester. The placenta was collected at the time of onset (10 days after symptoms) for investigation of CHIKV infection. Newborn serum was nonreactive IgG and IgM on day 10 (result provided by hospital).
– Case 2: 26-year-old patient, 7 weeks pregnant (1^st^ trimester). In July 2018, she attended the Regional Center for Infectious Diseases (CRDI) at the Plantadores de Cana Hospital (Campos dos Goytacazes) reporting that he had started symptoms such as arthralgia, rash, fever and nausea for 4 days. At the time of consultation, she was afebrile, anicteric and acyanotic, with blood pressure of 120 × 80 mmHg. Serum was collected for serology tests (1^st^ sample was collected on July 17, 2018—IgM reactive; 2^nd^ sample was collected 10 days later—IgG reactive; both during 1^st^ trimester). The placenta was collected at delivery (40 weeks). The newborn was asymptomatic newborn, and not tested.
– Case 3: Patient aged 19 years. She attended the Gafrée and Guinle University Hospital (HUGG), Rio de Janeiro, at 12 weeks of gestation, reporting symptoms such as arthralgia, rash and fever, when the serum was positive for CHIKV (PCR and serology—IgM). At 20 weeks, she was admitted to the ICU with urinary sepsis. The placenta was collected later at delivery at 40 weeks’ gestation. There is no information about the serum of the newborn.
– Case 4: A 37-year-old patient compared to the Regional Center for Infectious Diseases (CRDI) at the Plantadores de Cana Hospital (Campos dos Goytacazes). She had symptoms at the time of delivery, in September 2018, when the placenta was collected (38 weeks). The newborn had non-reactive anti-CHIKV IgG and IgM on the first day of life (result provided by hospital).
– Case 5: A 28-year-old patient in the third trimester of pregnancy. Attended the Hospital Dr. Beda (Campos dos Goytacazes) in June 2019 for delivery (38 weeks and 4 days), in which serum was collected for serological tests (IgM reactive; IgG non-reactive). She reported symptoms such as severe arthralgia, rash, fever and headache 2 days before delivery.

### Sample collection

Blood samples from the pregnant women were collected upon onset of symptoms. Placenta samples were collected and fixed in formalin (10%) upon delivery. Samples were collected at the Plantadores de Cana Hospital, Gaffrée and Guinle University Hospital, and the Doctor Beda General Hospital. Samples of term placentas from healthy donors served as controls.

### Serological diagnosis of chikungunya

Serological diagnosis was performed on serum samples from PW (controls and cases) using commercial kits, according to the manufacturer’s protocol. Anti-CHIKV IgM and IgG antibodies were detected using Anti-CHIKV IgM and IgG ELISA kits, respectively (Euroimmun, Lubeck, Germany).

### Viral RNA extraction and RT-qPCR for chikungunya virus in placental samples

RNA was extracted from formalin-fixed paraffin-embedded (FFPE) placental samples (collected at term—from the 37th week of pregnancy—during the delivery) using the PureLink™ FFPE RNA Isolation Kit (Invitrogen, Carlsbad California, USA) following the manufacturer’s instructions and stored at-70°C. RT-qPCR of CHIKV RNA was performed as described elsewhere (Lanciotti et al., 2007) using the ABI Prism® 7,500 Sequence Detection System (Applied Biosystems, Foster City, California, USA).

### Histopathological analysis

Placental samples fixed in formalin (10%) were sectioned, dehydrated in ethanol, clarified in xylene-impregnated paraffin, and embedded in paraffin resin. Tissue was then sectioned into 5 μm slides and incubated for 90 min at 60°C to melt the paraffin. Slides were then deparaffinized in xylene and rehydrated with decreasing concentrations of ethanol (100 to 70%). The slides were subsequently stained with hematoxylin and eosin and visualized by light microscopy (Olympus BX 53F, Japan). Digital images of main histological features were obtained using Image-Pro Plus software version 4.5.

### Immunohistochemistry procedure

The paraffin-embedded tissues (4 μm thick) were incubated for 90 min at 60°C, deparaffinized in xylene, and rehydrated with ethanol. Antigen retrieval was performed by heating the tissue in the presence of citrate buffer (pH 6.0). Tissues sections were then incubated with 3% hydrogen peroxide in methanol for 10 min to block endogenous peroxidase and then rinsed in Tris–HCl (pH 7.4). Slides were then rinsed in Protein Blocker solution (ScyTek, Logan, Utah, USA) for 10 min to reduce non-specific binding. Samples were incubated with the following primary antibodies: polyclonal anti-CHIKV mouse hyperimmune ascites fluids diluted 1:700; and monoclonal anti-human against: CD8 (DAKO Cytomation, USA, diluted 1:100); CD163 (Abcam, United Kingdom, 1:200); IFN-γ (R&D Systems, USA, diluted 1:30); TNF-α (Abbiotec, USA, diluted 1:200); TGF-β (Santa Cruz, USA, diluted 1:300) and IL-10 (R&D Systems, USA, diluted 1:100) and at 4°C overnight. Subsequent incubations were performed with the Two-Step Polymer Immunohistoprobe Plus (Redwood, California, USA): Amplifier for Mouse & Rabbit IgG for 15 min and HRP Polymer Detector at room temperature for 15 min. Samples were exposed to diaminobenzidine (ScyTek, Logan, Utah, United States), and Mayer’s hematoxylin (Dako, Palo Alto, California, USA) was used to counterstain. Sections were analyzed with an Olympus BX 53 microscope and images were acquired using a coupled Olympus DP72 camera.

### Quantification analysis

To quantify positive cells, images from 20 random fields for each specific primary antibody (3 negative controls and 5 positive CHIKV cases) were acquired at 1,000× magnification, in an Olympus BX 53F microscope, using the software Image Pro version 7. The number of positive cells was quantified (manually counted) in each of the 20 fields, and the mean per placenta was calculated. Representative images are provided on the boards. Three placenta controls (negative for CHIKV) collected between the 28th and 40th week of pregnancy were included.

### Statistical analysis

Data were analyzed with GraphPad Prism software v 6.0 (GraphPad Software, San Diego, California, USA). Cases and controls were compared using a Mann–Whitney test with a significance level of 0.05.

## Results

### Serological and molecular tests

Case 1 presented anti-CHIKV IgM and IgG antibodies; whereas cases 2 and 3 presented only anti-CHIKV IgG or anti-CHIKV IgM, respectively. It was not possible to perform the serology of case 4 as the serum sample was not available. Moreover, CHIKV RNA was detected by RT-qPCR in cases l, 2, 3 and 4 in FFPE placenta samples; and in the serum of case 5. All experiments were performed in duplicate (Ct1 and Ct 2). Serum samples of control pregnant women were tested for serology during the 3^rd^ trimester, as well as cases 1 and 5. Cases 2 and 3 were tested during the 1^st^ trimester. Chikungunya RT-qPCR was performed in FFPE placenta samples of 3^rd^ trimester, which were collected at the delivery (Table 1).

**Table 1:** Serological and molecular tests.

Samples	Chikungunya serology		Chikungunya RT-qPCR
	IgM	IgG	Result
Control 1	No	No	Negative
Control 2	No	No	Negative
Control 3	No	No	Negative
Case 1	Yes	Yes	Positive
Case 2	No	Yes	Positive
Case 3	Yes	No	Positive
Case 4	Serology was not performed	Serology was not performed	Positive
Case 5	Yes	No	Positive
Case 5^a^	Yes	No	Positive

Serology was performed using the serum of pregnant women, and the molecular tests were performed using FFPE placenta collected at the delivery. Serum samples: Controls, cases 1 and 5 were tested for serology during the 3^rd^ trimester; cases 2 and 3 were tested during the 1^st^ trimester. Chikungunya RT-qPCR was performed in FFPE placenta samples of 3^rd^ trimester.

a Newborn’s serum.

### Histopathological analysis

**Figure 1:**
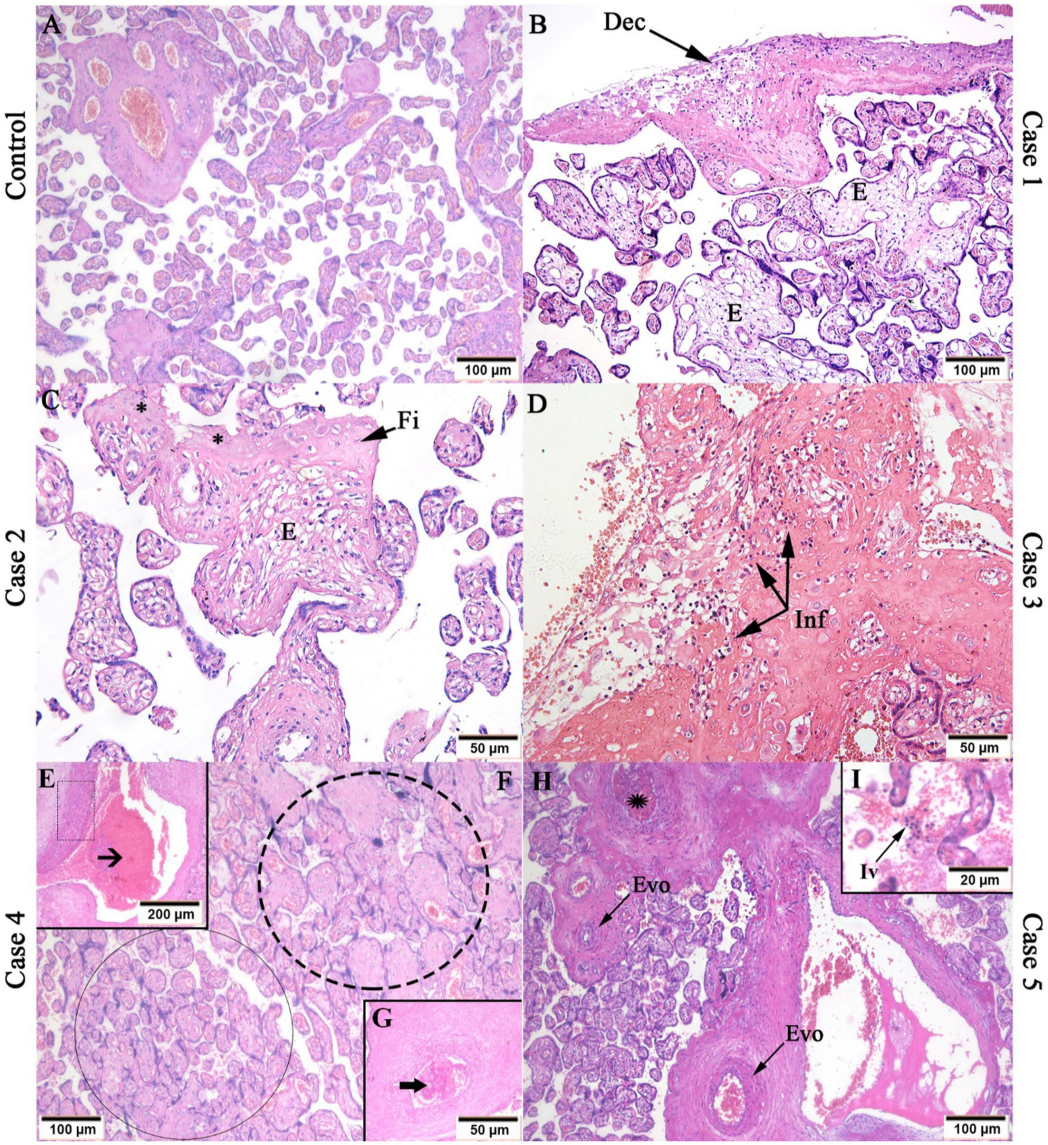
Histopathological analysis of CHIKV infected placenta tissues. **(A)** Control placenta tissue with regular aspects; **(B)** Deciduitis (Dec) and villous edema **(E)** in case 1; **(C)** Fibrin deposits (Fi), edema (E) and necrosis (*) in case 2; **(D)** Inflammatory infiltrate (Inf) in case 3; **(E)** Stem vessel vascular intramural fibrinoid deposition (dotted rectangle) and subocclusive thrombosis (black arrow); **(F)** avascular villi with stromal fibrosis (black circle) and villous stromal-vascular karyorrhexis (dotted circle); **(G)** Stem vessel with occlusive thrombosis (black thick arrow) in case 4; **(F)** Stem vessel obliteration/fibromuscular sclerosis (Evo) and thrombosis (black star); intervillositis (Iv) in case 5. Magnification: 5× **(E)**, 10× **(A,B,F,H)**, 20× **(C,D,G)**, 40× **(I)**.

In case 1, deciduitis (inflammatory infiltrates in the decidua), and edema in the chorionic villi (Figure 1B) were found; as well as areas of necrosis, fibrin deposits and villous edema in case 2 (Figure 1C). Moderate inflammatory infiltrates were observed in the decidua in case 3 (Figure 1D). Stem vessel with subocclusive (Figure 1E) and occlusive (Figure 1G) and thrombosis were seen in case 4, as well as in case 5 (Figure 1H). Intervillositis was observed in case 5 (Figure 1I). In case 5, a group of avascular villi with stromal fibrosis and karyorrhexis was noted (Figure 1F). As expected, the control placenta tissue did not exhibit alterations (Figure 1A).

### Chikungunya antigen detection in placental tissues

**Figure 2:**
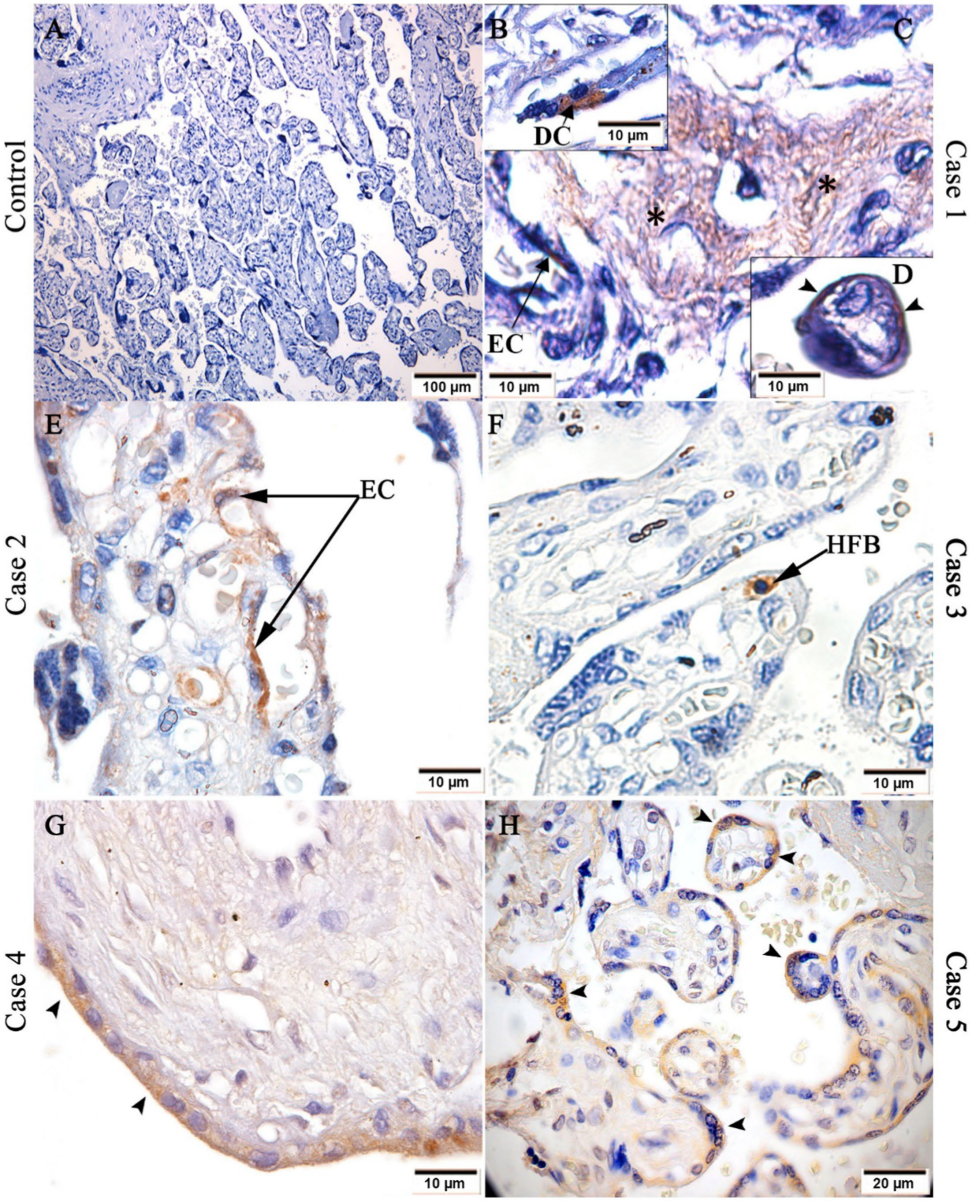
Detection of CHIKV antigen in placental tissue. **(A)** Control placenta with no CHIKV antigen detection; CHIKV antigen detection in: **(B)** decidual cell, **(C)** endothelial cell and stroma (black asterisks) of chorionic villi and **(D)** trophoblastic cells of case 1, **(E)** endothelial cells (EC) of case 2; **(F)** Hofbauer cell (HFB) of case 3; **(G)** trophoblastic cells of case 4; and **(H)** trophoblastic cells of case 5. Magnification: 10× **(A)**, 40× **(H)**, 100× **(B–G)**.

Using the immunohistochemistry (IHC) technique, it was possible to detect the CHIKV viral antigen in case 1, in decidual cells (Figure 2B), endothelium of fetal cells, stroma villi (Figure 2C) and trophoblastic cells (Figure 2D). In addition, the antigen was present in endothelial cells of case 2 (Figure 2E), in Hofbauer cells of case 3 (Figure 2F) and in trophoblastic cells of cases 4 (Figure 2G) and 5 (Figure 2H). As expected, no viral antigen was detected in the control placental tissue (Figure 2A).

### Cellularity in CHIKV-infected placenta

**Figure 3:**
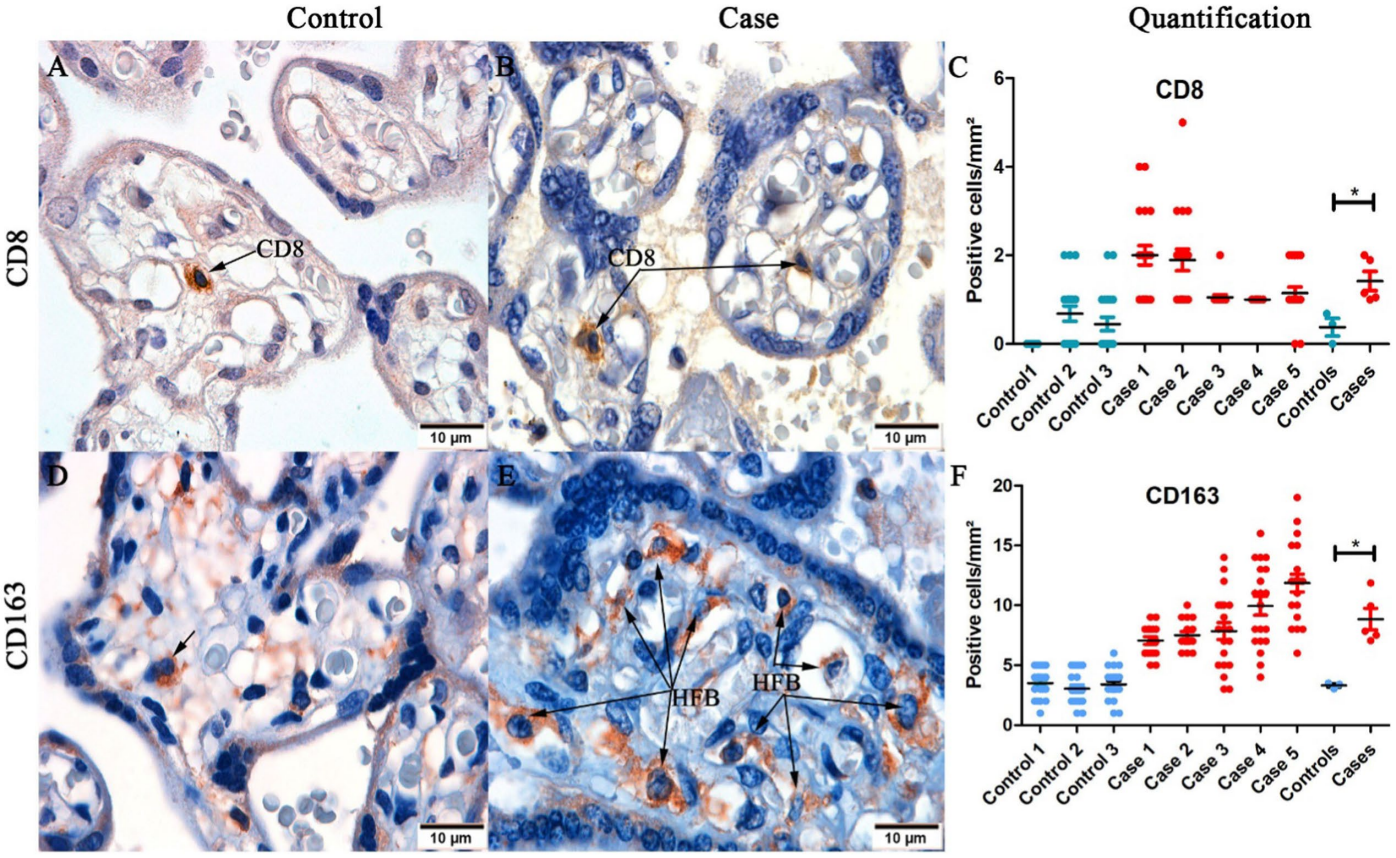
Representative images of detection of CD8^+^ and CD163^+^ cells in placental tissue. **(A)** Control placenta with low detection of CD8^+^ T cells; **(B)** Infected placenta with high detection of CD8+ T cells; **(C)** Quantification of CD8+ T cells in control and infected placentas; **(D)** Control placenta with low detection of CD163^+^ cells; **(E)** Infected placenta with high detection of CD163^+^ cells **(F)** Quantification and statistical analysis of CD163^+^ cells between control and infected placentas. (*p < 0.05). Data are represented as mean ± SDM. Magnification: 100× **(A–D)**.

In order to find whether cellularity of the CHIKV-infected placenta was altered, the presence of CD8^+^ and CD163^+^ cells were investigated. In control placenta, there was low expression of these markers (Figures 3A,D) compared to infected placenta (Figures 3B,E). There were significant differences between cases and controls in CD8 expression (Figure 3C) and CD163 expression (Figure 3F).

### Cytokine expression in CHIKV-infected placentas

**Figure 4:**
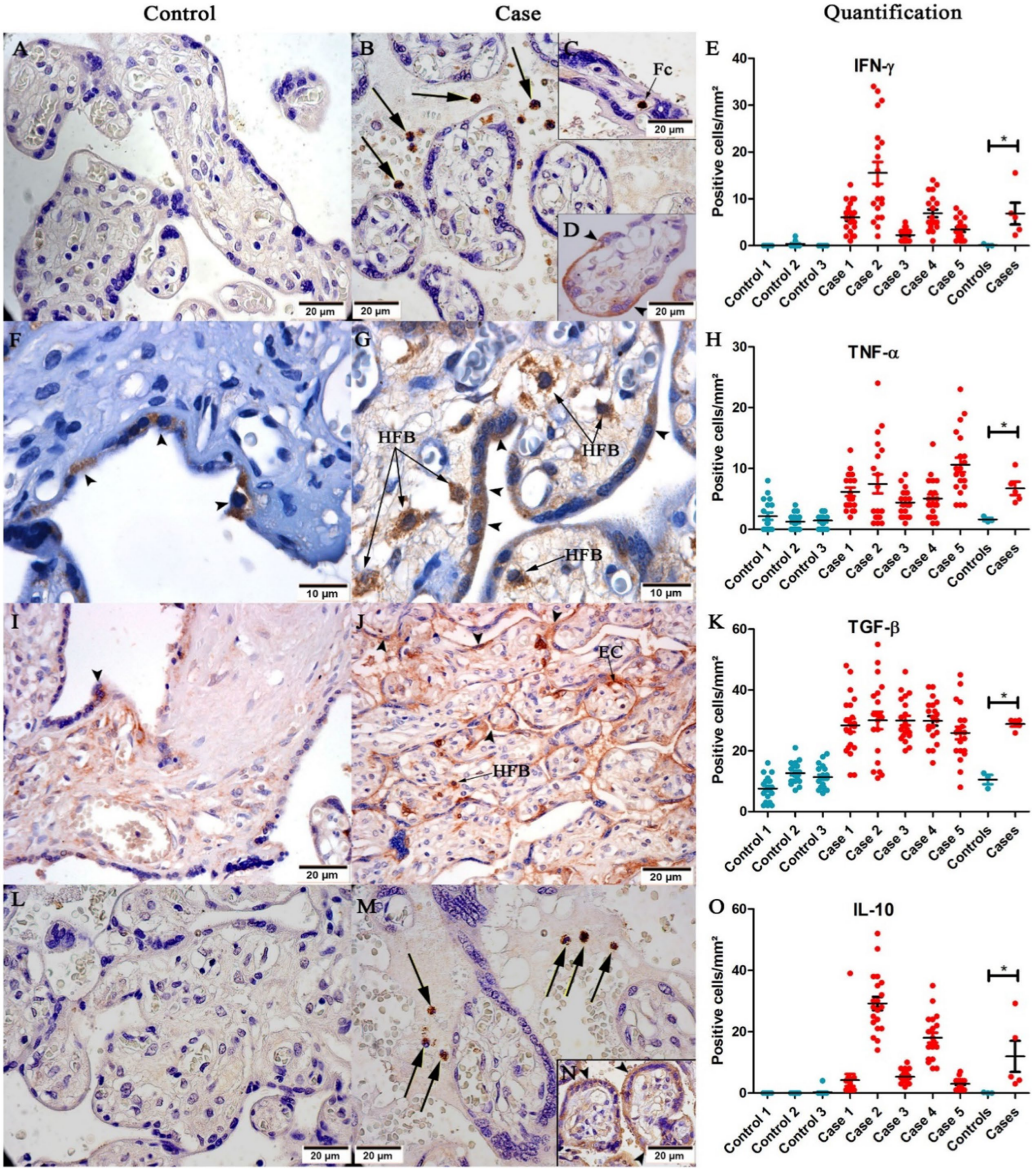
Representative images of cytokine detection in placental tissue. **(A)** No IFN-γ expression in control placenta; **(B)** IFN-γ expression in inflammatory cells (black arrows), **(C)** fetal cells (Fc), **(D)** and trophoblast cells (➤) in placental case; **(F)** Low TNF-α expression in trophoblast cells (➤) of the control placenta; **(G)** TNF-α expression in Hofbauer cells (HFB) in placental case; **(I)** Low expression of TGF-β in trophoblast cells (➤) in the control placenta; **(J)** TGF-β expression in trophoblast cells (➤), endothelial cells (EC) and Hofbauer cells (HFB); **(L)** No expression of IL-10 in placental case; **(M)** IL-10 expression in inflammatory cells (black arrows), and **(N)** trophoblast cells (➤). Quantification of **(E)** IFN-γ; **(H)** TNF-α; **(K)** TGF-β; and **(O)** IL-10 expression. (*p < 0.05). Data are represented as mean ± SDM. Magnification: 40× **(A,B,C,D,I,J,L,M,N)**, 100× **(F,G)**.

Cytokine expression in the tissue was assessed to investigate the immune response profile to CHIKV infection. To this end, pro-inflammatory (IFN-γ and TNF-α) and anti-inflammatory (TGF-β and IL-10) cytokines were analyzed. IFN-γ was expressed by inflammatory cells in the intervillous space (Figure 4B), fetal cells inside fetal capillaries in chorionic villi (Figure 4C), and trophoblastic cells (Figure 4D). IFN-γ had low or no expression in controls (Figure 4A). TNF-α was expressed in trophoblastic cells in the control placenta (Figure 4F), and in Hofbauer cells in infected placenta (Figure 4G). TGF-β was found in trophoblast cells (Figure 4I), Hofbauer cells and endothelial cells (Figure 4J); and IL-10 in inflammatory cells in the intervillous space (Figure 4M) and trophoblast cells (Figure 4N). IL-10 had low or no expression in controls (Figure 4L). Quantification was performed to compare control and CHIKV-infected placenta expression. Cytokines expression evidenced statistical difference between the two groups (controls and cases): IFN-γ (Figure 4E), TNF-α (Figure 4H), TGF-β (Figure 4K), and IL-10 (Figure 4O).

## Discussion

The exact mechanism of CHIKV transmission to the fetus and the possible role of the placenta in this process remain unknown. Herein we analyzed placental tissues from five cases of CHIKV infection during the 1^st^, 2^nd^ and 3^rd^ trimesters. FFPE placental tissues were positive for CHIKV by real time RT-PCR in cases 1, 2, 3, and 4. In case 5, real time RT-PCR was performed only on the serum (mother and newborn) with positives results, evidencing the presence of CHIKV genome.

The serology was performed to analyze the presence of anti-CHIKV antibodies. Usually, IgM antibody is detected during the acute phase (≤7 days post symptom onset) of CHIKV infection, indicating recent infection; however could persist up to 3 months (Andrew et al., 2022). IgG antibodies are used to indicate late infection, since these antibodies are detected mainly in samples of convalescent phase (>7 days post symptom onset; Andrew et al., 2022). For example, the serum of case 2 was collected at delivery and evidenced the presence of IgG antibodies only, displaying a late infection, during the first trimester of gestation.

Regarding histopathological alterations, we found features of fetal vascular malperfusion: such as (1) stem vessel obliteration, with intramural fibrinoid deposition and also fibromuscular sclerosis, defined as thickening of the vessel wall, that results in partial or complete obliteration of the vascular lumen (Khong et al., 2016); and (2) occlusive thrombosis, a blood clot in the fetal villi vessel which blocks the blood passage, impairing exchanges between mother and fetus (Kraus, 2013). These changes were also reported in placental samples in cases of maternal hypertension and diabetes mellitus (Aldahmash et al., 2021), SARS-CoV-2 (Rebutini et al., 2021), dengue virus (DENV) (Fernandes Ribeiro et al., 2017) and other congenital infections (Stanek et al., 2011; Heider, 2017; Roberts and Torous, 2022).

We also noted villous edema and fibrinoid deposition, which were already described by Sandoval (Sandoval, 2016), and in other arboviruses infections such as DENV (Nunes et al., 2016, 2019; Fernandes Ribeiro et al., 2017) and zika virus (ZIKV; Rabelo et al., 2018, 2020). Villous edema is a pathological change related to hypoxia (Fernandes Ribeiro et al., 2017). Hypoxia in early pregnancy can alter trophoblastic differentiation and placental establishment; and in late gestational hypoxia includes changes in mitochondrial functions, endoplasmic reticulum stress, hormone production, nutrition and secretion of angiogenic factors; which depends on the extent of hypoxia (Colson et al., 2021). The increase of perivillous fibrin seems to occur in cases of trophoblastic necrosis (Chen and Roberts, 2018), which was observed in case 2. Depending on the extent of the fibrin deposition, obstruction of maternal flow may be observed, causing fetal consequences (Chen and Roberts, 2018). Mononuclear inflammatory cell infiltration in the decidua (deciduitis) and in the intervillous space (intervillositis) are chronic inflammatory lesions and have also observed as in other viral infections, such as ZIKV (Chimelli et al., 2017; Zanluca et al., 2018), DENV (Fernandes Ribeiro et al., 2017) and SARS-CoV-2 (Vivanti et al., 2020; Leal et al., 2021; Marton et al., 2021). In addition, placental exhibited dystrophic calcification, a deposition of calcium phosphate crystals in damaged areas, with necrotic portions or decreased circulation. This type of observation was noted also in ZIKV-infected placenta (Azevedo et al., 2018; Rabelo et al., 2020).

The use of the IHC technique facilitated immunolocalization of CHIKV antigen in decidual cells, Hofbauer cells, endothelial cells, stroma of chorionic villi, trophoblast cells and in cells of the intervillous space. Here, we have representative images of CHIKV^+^ cells in each case; however, we cannot relate the period of infection to the cell type positive for the virus; furthermore, the sample size is small to define it. These target cells are also positive in other arboviruses infections, such as DENV (Nunes et al., 2016; Fernandes Ribeiro et al., 2017) and ZIKV (Rabelo et al., 2017, 2020).

CD8^+^ cell expression was increased in infected placentas compared to controls, particularly in cases 1 and 2, consistent with the inflammatory infiltrate areas observed in histopathological findings. The recruitment of CD8+ T cells to tissue typically occurs due to pathogenic infection and contributes to IFN-γ and TNF-α production (Halle et al., 2017). Increase of these cells were reported also in cases of pregnancies complicated by pre-eclampsia, fetal growth restriction and small-for-gestational age (Lager et al., 2020).

CD163, which served as a macrophage activation marker, and Hofbauer cells (fetal-placental macrophages) were increased in infected placental tissues compared to the control. Hyperplasia of Hofbauer cells was also reported in ZIKV-infected placenta (Schwartz, 2017; De Noronha et al., 2018; Rabelo et al., 2020), gestational diabetes mellitus (Barke et al., 2018) and glucocorticoid administration (Tang et al., 2013). Hyperplasia of Hofbauer cells is a pathologic condition reported in ascending infections, TORCH infections, and villitis of unknown etiology (Schwartz, 2017). It contributes to the immunopathogenesis of the disease, since it plays a role in the production of inflammatory mediators; in addition to being a site of viral replication. In ZIKV studies, authors suggest that these cells could play a role in the dissemination of ZIKV to the fetus, as they are mobile and occur in perivascular locations (Simoni et al., 2017). Similarly, we found that CHIKV was able to infect Hofbauer cells, so it could be a pathway through which the virus reaches the fetus; however, this mechanism needs further investigation.

As well as histopathological changes, the infiltration of CD8^+^ T cells, fetal macrophages (Hofbauer cells) and several inflammatory cells into the chorionic villi may happen at any stage of pregnancy; which will culminate in complicated gestation or bad outcomes depending on the extension.

In addition to cellularity, cytokine profiles were analyzed through the immunohistochemistry. IFN-γ and TNF-α are pro-inflammatory cytokines, expression of which was increased in infected placentas compared to controls, and was expressed mainly by trophoblast cells, Hofbauer cells and inflammatory cells (in intervillous space and inside fetal capillaries); contributing to a pro-inflammatory environment. High expression of TNF-α in these cell types was also reported in DENV (Nunes et al., 2019) and ZIKV (Rabelo et al., 2020) infections.

In addition to the increase of pro-inflammatory cytokines, we also found increase of anti-inflammatory cytokines; perhaps as an attempt to contain the local inflammatory process without threatening the pregnancy. Anti-inflammatory cytokines such as IL-10 and transforming growth factor (TGF-β) are typically increased primarily in the second trimester, and decrease during the first and third trimesters (Bränn et al., 2019; Meyyazhagan et al., 2022). For the two cytokines we noted statistical difference between controls and cases. This increase has also been reported in cases of pre-eclampsia (Lyall et al., 2001) and molar lesions (Xuan et al., 2007); which could result in inhibition of trophoblast cell migration and invasion (Yang et al., 2021). High IL-10 expression was also reported in ZIKV-infected HTR8 (Luo et al., 2018). Such an increase was also observed in placental cytotrophoblast in Cytomegalovirus infection, associated with reduced matrix metalloproteinase 9 activity, which contributes to impaired cytotrophoblast remodeling of the uterine vasculature and consequently restricted fetal growth (Yamamoto-Tabata et al., 2004). Moreover, TGF-β and IL-10 are associated with tissue repair, healing and angiogenesis (Schliefsteiner et al., 2020).

CHIKV vertical transmission is known to occur, although is a rare event. Here, in all placentas we found histopathological and immunological changes, in addition to CHIKV antigen detection. However, vertical transmission was confirmed in only one case. Our findings highlight the importance of avoiding infections during pregnancy − in this case, Chikungunya − since modifications of the placenta can interfere with the functioning of this organ, and culminate in unfavorable outcomes for the pregnant women and fetus.

## Data availability statement

The original contributions presented in the study are included in the article, further inquiries can be directed to the corresponding author.

## Ethics statement

The studies involving human participants were reviewed and approved by Ethics Committee of the Oswaldo Cruz Foundation. The patients/participants provided their written informed consent to participate in this study.

## Author contributions

MP and NS: conceptualization. NS, KR, TMS, ML, and PN: methodology. MP, NS, LF, and EA-P: formal analysis. PB and MP: investigation. MP, Fd-S, CB-d-O, and RB-d-O: resources. FF, LJ-d-S, RF, APS, and LD: data curation. NS: writing—original draft preparation. KR, Fd-S, PB, and EA-P: writing—review and editing. EA-P, Fd-S, and MP: supervision. MP and Fd-S: project administration. MP, Fd-S, and PB: funding acquisition. All authors contributed to the article and approved the submitted version.

## Funding

This research was funded by Fundação Carlos Chagas Filho de Amparo à Pesquisa do Estado do Rio de Janeiro, grants numbers: E-26/210.400/2019 (MP), E-26/202.003/2016 (FS), E-26/202.659/2019 (FS), E-26/211.569/2019 (FS), E-26/211.565/2019 (PB), E-26/202.862/2018 (PB) and by Conselho Nacional de Desenvolvimento Científico e Tecnológico (CNPq), grant numbers: 302462/2018–0 (FS) and 307282/2017-1 (PB); and DECIT/25000.072811/2016-19 (PB).

## Conflict of interest

The authors declare that the research was conducted in the absence of any commercial or financial relationships that could be construed as a potential conflict of interest.

## Publisher’s note

All claims expressed in this article are solely those of the authors and do not necessarily represent those of their affiliated organizations, or those of the publisher, the editors and the reviewers. Any product that may be evaluated in this article, or claim that may be made by its manufacturer, is not guaranteed or endorsed by the publisher.

## References

[ref1] Aldahmash W. M. Alwasel S. H. Aljerian K. (2021). Gestational diabetes mellitus induces placental vasculopathies. Environ. Sci. Pollut. Res. 29, 19860–19868. doi: 10.1007/S11356-021-17267-Y, PMID: 34725760

[ref2] Andrew A. Navien T. N. Yeoh T. S. Citartan M. Mangantig E. Sum M. S. H.. (2022). Diagnostic accuracy of serological tests for the diagnosis of chikungunya virus infection: a systematic review and meta-analysis. PLoS Negl. Trop. Dis. 16:e0010152. doi: 10.1371/JOURNAL.PNTD.0010152, PMID: 35120141 PMC8849447

[ref3] Azevedo R. S. S. Araujo M. T. Oliveira C. S. Filho A. J. M. Nunes B. T. D. Henriques D. F.. (2018). Zika virus epidemic in Brazil. II. Post-mortem analyses of neonates with microcephaly, stillbirths, and miscarriage. J. Clin. Med. 7:496. doi: 10.3390/JCM7120496 30487475 PMC6306831

[ref4] Barke T. L. Goldstein J. A. Sundermann A. C. Reddy A. P. Linder J. E. Correa H.. (2018). “Gestational diabetes mellitus is associated with increased CD163 expression and iron storage in the placenta,” in Am. J. Reprod. Immunol., vol. 80, e13020. 29984475 10.1111/aji.13020 PMC6193471

[ref5] Bränn E. Edvinsson Å. Rostedt Punga A. Sundström-Poromaa I. Skalkidou A. (2019). Inflammatory and anti-inflammatory markers in plasma: from late pregnancy to early postpartum. Sci. Rep. 9, 1863–1810. doi: 10.1038/s41598-018-38304-w, PMID: 30755659 PMC6372606

[ref6] CGVS, C. G. de A. E. de V. em S (2019) ‘Área Programática, Regiões Administrativas e Bairros’. Available at: http://www.rio.rj.gov.br/dlstatic/10112/10829614/4293007/Chik2019mes.pdf (Accessed May 11, 2022).

[ref7] Chen A. Roberts D. J. (2018). Placental pathologic lesions with a significant recurrence risk – what not to miss! APMIS 126, 589–601. doi: 10.1111/APM.12796, PMID: 29271494

[ref8] Chimelli L. Melo A. S. O. Avvad-Portari E. Wiley C. A. Camacho A. H. S. Lopes V. S.. (2017). The spectrum of neuropathological changes associated with congenital Zika virus infection. Acta Neuropathol. 133, 983–989. doi: 10.1007/s00401-017-1699-5 28332092

[ref9] Colson A. Sonveaux P. Debiève F. Sferruzzi-Perri A. N. (2021). Adaptations of the human placenta to hypoxia: opportunities for interventions in fetal growth restriction. Hum. Reprod. Update 27, 531–569. doi: 10.1093/HUMUPD/DMAA053, PMID: 33377492

[ref10] de Noronha L. Zanluca C. Burger M. Suzukawa A. A. Azevedo M. Rebutini P. Z.. (2018). Zika virus infection at different pregnancy stages: Anatomopathological findings, target cells and viral persistence in placental tissues. Front. Microbiol. 9:2266. doi: 10.3389/FMICB.2018.02266/BIBTEX, PMID: 30337910 PMC6180237

[ref11] Escobar L. E. Qiao H. Peterson A. T. (2016). Forecasting chikungunya spread in the Americas via data-driven empirical approaches. Parasit. Vectors 9, 1–12. doi: 10.1186/S13071-016-1403-Y/FIGURES/6 26928307 PMC4772319

[ref12] Fernandes Ribeiro C. Lopes V. G. S. Brasil P. Pires A. R. C. Rohloff R. Nogueira R. M. R. (2017). ‘Dengue infection in pregnancy and its impact on the placenta’. Int J Infect Dis. 55, 109–112. 10.1016/j.ijid.2017.01.002 28088588

[ref13] Gérardin P. Barau G. Michault A. Bintner M. Randrianaivo H. Choker G.. (2008). Multidisciplinary prospective study of mother-to-child chikungunya virus infections on the island of La Réunion. PLoS Med. 5:e60. doi: 10.1371/journal.pmed.0050060, PMID: 18351797 PMC2267812

[ref14] Grivard P. le Roux K. Laurent P. Fianu A. Perrau J. Gigan J.. (2007). Molecular and serological diagnosis of chikungunya virus infection. Pathol. Biol. 55, 490–494. doi: 10.1016/j.patbio.2007.07.002 17920211

[ref15] Halle S. Halle O. Förster R. (2017). Mechanisms and dynamics of T cell-mediated cytotoxicity in vivo. Trends Immunol. 38, 432–443. doi: 10.1016/J.IT.2017.04.002, PMID: 28499492

[ref16] Heider A. (2017). Fetal Vascular Malperfusion. Arch. Pathol. Lab. Med. 141, 1484–1489. doi: 10.5858/ARPA.2017-0212-RA 29072954

[ref17] Higashi N. Matsumoto A. Tabata K. Nagatomo Y. (1967). Electron microscope study of development of chikungunya virus in green monkey kidney stable (VERO) cells. Virology 33, 55–69. doi: 10.1016/0042-6822(67)90093-1, PMID: 6039963

[ref18] Khong T. Y. Mooney E. E. Ariel I. Balmus N. C. M. Boyd T. K. Brundler M. A.. (2016). Sampling and definitions of placental lesions: Amsterdam placental workshop group consensus statement. Arch. Pathol. Lab. Med. 140, 698–713. doi: 10.5858/ARPA.2015-0225-CC, PMID: 27223167

[ref19] Kraus F. T. (2013). Fetal thrombotic vasculopathy: perinatal stroke, growth restriction, and other sequelae. Surg. Pathol. Clin. 6, 87–100. doi: 10.1016/J.PATH.2012.10.001, PMID: 26838704 PMC7102867

[ref20] Lager S. Sovio U. Eddershaw E. Linden M. W. Yazar C. Cook E.. (2020). Abnormal placental CD8+ T-cell infiltration is a feature of fetal growth restriction and pre-eclampsia. J. Physiol. 598, 5555–5571. doi: 10.1113/JP279532, PMID: 32886802

[ref21] Lanciotti R. S. Kosoy O. L. Laven J. J. Panella A. J. Velez J. O. Lambert A. J.. (2007). Chikungunya virus in US travelers returning from India, 2006. Emerg. Infect. Dis. 13, 764–767. doi: 10.3201/eid1305.070015, PMID: 17553261 PMC2738459

[ref22] Leal C. R. V. Maciel R. A. M. Corrêa Júnior M. D. (2021). SARS-CoV-2 infection and placental pathology Infecção por SARS-CoV-2 e patologia placentária. Rev. Bras. Ginecol. Obstet. 43, 474–479. doi: 10.1055/S-0041-1730291, PMID: 34077991 PMC10411154

[ref23] Lenglet Y. Barau G. Robillard P. Y. Randrianaivo H. Michault A. Bouveret A.. (2006). Chikungunya infection in pregnancy: evidence for intrauterine infection in pregnant women and vertical transmission in the parturient. Survey of the Reunion Island outbreak. J. Gynecol. Obstet. Biol. Reprod. (Paris) 35, 578–583. doi: 10.1016/S0368-2315(06)76447-X, PMID: 17003745

[ref24] Lumsden W. H. R. (1955). An epidemic of virus disease in Southern Province, Tanganyika territory, in 1952–1953 II. General description and epidemiology’. Transact. R. Soc. Trop. Med. Hyg. 49, 33–57. doi: 10.1016/0035-9203(55)90081-X, PMID: 14373835

[ref25] Luo H. Winkelmann E. R. Fernandez-Salas I. Li L. Mayer S. V. Danis-Lozano R.. (2018). Zika, dengue and yellow fever viruses induce differential anti-viralimmune responses in human monocytic and first trimester trophoblastcells. Antivir. Res. 151, 55–62. doi: 10.1016/J.ANTIVIRAL.2018.01.003, PMID: 29331320 PMC5844857

[ref26] Lyall F. Simpson H. Nicola Bulmer J. Barber A. Courtenay Robson S. (2001). Transforming growth factor-β expression in human placenta and placental bed in third trimester Normal pregnancy, preeclampsia, and fetal growth restriction. Am. J. Pathol. 159, 1827–1838. doi: 10.1016/S0002-9440(10)63029-5, PMID: 11696443 PMC1867050

[ref27] Lyra P. Campos G. Bandeira I. Sardi S. Costa L. Santos F.. (2016). Congenital chikungunya virus infection after an outbreak in Salvador, Bahia, Brazil. AJP Rep. 06, e299–e300. doi: 10.1055/S-0036-1587323, PMID: 27555980 PMC4993616

[ref28] Marton T. Hargitai B. Hunter K. Pugh M. Murray P. (2021). Massive Perivillous fibrin deposition and chronic histiocytic Intervillositis a complication of SARS-CoV-2 infection. Pediatr. Dev. Pathol. 24, 450–454. doi: 10.1177/10935266211020723, PMID: 34082613

[ref29] Mavalankar D. Shastri P. Bandyopadhyay T. Parmar J. Ramani K. V. (2008). Increased mortality rate associated with chikungunya epidemic, Ahmedabad, India. Emerg. Infect. Dis. 14, 412–415. doi: 10.3201/eid1403.070720, PMID: 18325255 PMC2570824

[ref30] Meyyazhagan A. Kuchi Bhotla H. Pappuswamy M. Tsibizova V. al Qasem M. di Renzo G. C. (2022). Cytokine see-saw across pregnancy, its related complexities and consequences. Int. J. Gynecol. Obstet. doi: 10.1002/IJGO.14333, PMID: 35810391

[ref31] Nunes P. Nogueira R. Coelho J. Rodrigues F. Salomão N. José C.. (2019). A stillborn multiple organs’ investigation from a maternal denv-4 infection: histopathological and inflammatory mediators characterization. Viruses 11:319. doi: 10.3390/v11040319, PMID: 30986974 PMC6521294

[ref32] Nunes P. C. G. Paes M. V. de Oliveira C. A. B. Soares A. C. G. de Filippis A. M. B. Lima M. R. Q.. (2016). Detection of dengue NS1 and NS3 proteins in placenta and umbilical cord in fetal and maternal death. J. Med. Virol. 88, 1448–1452. doi: 10.1002/jmv.24479, PMID: 26792253

[ref33] Powers A. M. Brault A. C. Shirako Y. Strauss E. G. Kang W. L. Strauss J. H.. (2001). Evolutionary relationships and systematics of the alphaviruses. J. Virol. 75, 10118–10131. doi: 10.1128/jvi.75.21.10118-10131.2001, PMID: 11581380 PMC114586

[ref34] Prata-Barbosa A. Cleto-Yamane T. L. Robaina J. R. Guastavino A. B. de Magalhães-Barbosa M. C. Brindeiro R. M.. (2018). Co-infection with Zika and chikungunya viruses associated with fetal death—a case report. Int. J. Infect. Dis. 72, 25–27. doi: 10.1016/j.ijid.2018.04.4320, PMID: 29738826

[ref35] Rabelo K. de Souza Campos Fernandes R. C. Souza L. J. Louvain de Souza T. Santos F. B. Guerra Nunes P. C.. (2017). Placental histopathology and clinical presentation of severe congenital Zika syndrome in a human immunodeficiency virus-exposed uninfected infant. Front. Immunol. 8:1704. doi: 10.3389/fimmu.2017.01704, PMID: 29270171 PMC5725436

[ref36] Rabelo K. de Souza L. J. Salomão N. G. Machado L. N. Pereira P. G. Portari E. A.. (2020). Zika induces human placental damage and inflammation. Front. Immunol. 11:2146. doi: 10.3389/FIMMU.2020.02146/BIBTEX, PMID: 32983175 PMC7490298

[ref37] Rabelo K. Souza L. J. Salomão N. G. Oliveira E. R. A. Sentinelli L. P. Lacerda M. S.. (2018). Placental inflammation and fetal injury in a rare Zika case associated with Guillain-Barré syndrome and abortion. Front. Microbiol. 9:1018. doi: 10.3389/fmicb.2018.01018, PMID: 29867903 PMC5964188

[ref38] Ramful D. Carbonnier M. Pasquet M. Bouhmani B. Ghazouani J. Noormahomed T.. (2007). Mother-to-child transmission of chikungunya virus infection. Pediatr. Infect. Dis. J. 26, 811–815. doi: 10.1097/INF.0b013e3180616d4f 17721376

[ref39] Rebutini P. Z. Zanchettin A. C. Stonoga E. T. S. Prá D. M. M. de Oliveira A. L. P. Dezidério F. S.. (2021). Association between COVID-19 pregnant women symptoms severity and placental morphologic features. Front. Immunol. 12:685919. doi: 10.3389/FIMMU.2021.685919, PMID: 34122449 PMC8187864

[ref40] Roberts D. J. Torous V. (2022). ‘Placental pathology’, reproductive and developmental. 3rd *Edn*. Toxicology, 1399–1420. doi: 10.1016/B978-0-323-89773-0.00069-2

[ref41] Robillard P. Y. Boumahni B. Gérardin P. Michault A. Fourmaintraux A. Schuffenecker I.. (2006). Transmission verticale materno-fœtale du virus chikungunya: Dix cas observés sur l’île de la Réunion chez 84 femmes enceintes. Presse Med. 35, 785–788. doi: 10.1016/S0755-4982(06)74690-5 16710146

[ref42] Salomão N. Brendolin M. Rabelo K. Wakimoto M. de Filippis A. M. dos Santos F.. (2021). Spontaneous abortion and chikungunya infection: pathological findings. Viruses 13:554. doi: 10.3390/v13040554, PMID: 33806252 PMC8067258

[ref43] Sandoval O. (2016). *The Placenta in a Case of Pregnant Woman Infected by Chikungunya Virus*.

[ref44] Schliefsteiner C. Ibesich S. Wadsack C. (2020). Placental Hofbauer cell polarization resists inflammatory cues in vitro. Int. J. Mol. Sci. 21:736. doi: 10.3390/IJMS21030736, PMID: 31979196 PMC7038058

[ref45] Schuffenecker I. Iteman I. Michault A. Murri S. Frangeul L. Vaney M. C.. (2006). Genome microevolution of chikungunya viruses causing the Indian Ocean outbreak. PLoS Med. 3:e263. doi: 10.1371/journal.pmed.0030263, PMID: 16700631 PMC1463904

[ref46] Schwartz D. A. (2017). Viral infection, proliferation, and hyperplasia of Hofbauer cells and absence of inflammation characterize the placental pathology of fetuses with congenital Zika virus infection. Arch. Gynecol. Obstet. 295, 1361–1368. doi: 10.1007/S00404-017-4361-5, PMID: 28396992 PMC5429341

[ref47] Secretaria de estado de saúde do Rio de Janeiro (2019) ‘BOLETIM EPIDEMIOLÓGICO ARBOVIROSES N^o^ 002/2019 CENÁRIO’. Available at: http://www.riocomsaude.rj.gov.br/Publico/MostrarArquivo.aspx?C=F%2BJ77ZiVqng%3D (Accessed May 11, 2022).

[ref48] Senanayake M. P. Senanayake S. M. Vidanage K. K. Gunasena S. Lamabadusuriya S. P. (2009). Vertical transmission in chikungunya infection. Ceylon Med. J. 54:47. doi: 10.4038/cmj.v54i2.865 19670548

[ref49] Shenoy S. Pradeep G. C. M. (2012). Neurodevelopmental outcome of neonates with vertically transmitted chikungunya fever with encephalopathy. Indian Pediatr. 49, 238–240. 22484743

[ref50] Silva L. A. Dermody T. S. (2017). Chikungunya virus: epidemiology, replication, disease mechanisms, and prospective intervention strategies. J. Clin. Invest. 127, 737–749. doi: 10.1172/JCI84417, PMID: 28248203 PMC5330729

[ref51] Simizu B. Yamamoto K. Hashimoto K. Ogata T. (1984). Structural proteins of chikungunya virus. J. Virol. 51, 254–258. doi: 10.1128/jvi.51.1.254-258.1984, PMID: 6726893 PMC254427

[ref52] Simoni M. K. Jurado K. A. Abrahams V. M. Fikrig E. Guller S. (2017). Zika Virus infection of Hofbauer cells. Am. J. Reprod. Immunol. 77. doi: 10.1111/AJI.12613 PMC5299062 27966815

[ref53] Stanek J. Sheridan R. M. le L. D. Crombleholme T. M. (2011). Placental fetal thrombotic vasculopathy in severe congenital anomalies prompting EXIT procedure. Placenta 32, 373–379. doi: 10.1016/J.PLACENTA.2011.02.002, PMID: 21435717

[ref54] Superintendência de Vigilância em Saúde (2018) Área Programática, Regiões Administrativas e Bairros. Available at: http://www.rio.rj.gov.br/dlstatic/10112/10829527/4267213/Chikmes 2018.pdf (Accessed May 11, 2022).

[ref55] Tang Z. Niven-Fairchild T. Tadesse S. Norwitz E. R. Buhimschi C. S. Buhimschi I. A.. (2013). Glucocorticoids enhance CD163 expression in placental Hofbauer cells. Endocrinology 154, 471–482. doi: 10.1210/EN.2012-1575, PMID: 23142809 PMC3529384

[ref56] Touret Y. Randrianaivo H. Michault A. Schuffenecker I. Kauffmann E. Lenglet Y.. (2006). Early maternal-fetal transmission of the chikungunya virus. Presse Med. 35, 1656–1658. doi: 10.1016/s0755-4982(06)74874-6 17086120

[ref57] Vivanti A. J. Vauloup-Fellous C. Prevot S. Zupan V. Suffee C. do Cao J.. (2020). Transplacental transmission of SARS-CoV-2 infection. Nat Commun. 11:3572. doi: 10.1038/S41467-020-17436-6, PMID: 32665677 PMC7360599

[ref58] Xuan Y. H. Choi Y. L. Shin Y. K. Ahn G. H. Kim K. H. Kim W. J.. (2007). Expression of TGF-beta signaling proteins in normal placenta and gestational trophoblastic disease. Histol. Histopathol. 22, 227–234. doi: 10.14670/HH-22.227, PMID: 17163397

[ref59] Yamamoto-Tabata T. McDonagh S. Chang H. T. Fisher S. Pereira L. (2004). Human cytomegalovirus Interleukin-10 downregulates metalloproteinase activity and impairs endothelial cell migration and placental Cytotrophoblast invasiveness in vitro. J. Virol. 78, 2831–2840. doi: 10.1128/JVI.78.6.2831-2840.2004, PMID: 14990702 PMC353759

[ref60] Yang D. Dai F. Yuan M. Zheng Y. Liu S. Deng Z.. (2021). Role of transforming growth factor-β1 in regulating fetal-maternal immune tolerance in Normal and pathological pregnancy. Front. Immunol. 12:3525. doi: 10.3389/FIMMU.2021.689181/BIBTEX PMC8438197 34531852

[ref61] Zanluca C. de Noronha L. Duarte dos Santos C. N. (2018). Maternal-fetal transmission of the zika virus: an intriguing interplay. Tissue Barriers 6:e1402143. doi: 10.1080/21688370.2017.1402143, PMID: 29370577 PMC5823548

